# Target Receptors of Regenerating Nerves: Neuroma Formation and Current Treatment Options

**DOI:** 10.3389/fnmol.2022.859221

**Published:** 2022-07-05

**Authors:** Feras Shamoun, Valentina Shamoun, Arya Akhavan, Sami H. Tuffaha

**Affiliations:** ^1^Peripheral Nerve Lab, Department of Plastic and Reconstructive Surgery, Johns Hopkins Hospital, Johns Hopkins University, Baltimore, MD, United States; ^2^Department of Biological Sciences, University of Toronto at Scarborough, Scarborough, ON, Canada

**Keywords:** neuroma, peripheral nerve regeneration, target receptors, targeted reinnervation, VDMT, RPNI, TMR

## Abstract

Neuromas form as a result of disorganized sensory axonal regeneration following nerve injury. Painful neuromas lead to poor quality of life for patients and place a burden on healthcare systems. Modern surgical interventions for neuromas entail guided regeneration of sensory nerve fibers into muscle tissue leading to muscle innervation and neuroma treatment or prevention. However, it is unclear how innervating denervated muscle targets prevents painful neuroma formation, as little is known about the fate of sensory fibers, and more specifically pain fiber, as they regenerate into muscle. Golgi tendon organs and muscle spindles have been proposed as possible receptor targets for the regenerating sensory fibers; however, these receptors are not typically innervated by pain fibers, as these free nerve endings do not synapse on receptors. The mechanisms by which pain fibers are signaled to cease regeneration therefore remain unknown. In this article, we review the physiology underlying nerve regeneration, the guiding molecular signals, and the target receptor specificity of regenerating sensory axons as it pertains to the development and prevention of painful neuroma formation while highlighting gaps in literature. We discuss management options for painful neuromas and the current supporting evidence for the various interventions.

## Introduction

Neuromas are bulbus swellings of abnormal and disorganized regeneration of predominantly unmyelinated nerve endings that form on the end of a peripheral nerve that is transected and left in discontinuity without timely repair (Cravioto and Battista, 1981). While some neuromas do not cause symptoms, they often produce disabling pain and poor quality of life in otherwise healthy individuals (Stokvis et al., 2010; Brogan and Kakar, 2013; Ellen and Renner, 2014). Patients suffering limb amputations are particularly susceptible, with rates ranging from 4.17 to 48% of patients with amputations (Penna et al., 2018). More than 185,000 people undergo amputations in the United States each year, and the total number of amputees is expected to more than double to nearly 3.6 million people by 2050 (Ziegler-Graham et al., 2008). Peripheral nerve injury places a significant burden on our healthcare system, with average care cost of approximately $47,000 per patient and therefore is an important area of investigation (Karsy et al., 2019).

Nerve injuries are classified according to the stage of nerve damage, which can determine functional prognosis and indication for nerve repair. The most commonly used classification is the Seddon-Sunderland classification system (Seddon, 1947; Sunderland and Williams, 1992). These classifications reflect the prognosis for nerve recovery (Chhabra et al., 2014; Lu et al., 2018), as detailed in Table 1. Neuromas arise from the disorganized regeneration of sensory axons in the absence of innervation targets, specifically the pain fiber subpopulation. If regenerating fibers find target tissue to innervate, neuroma formation is prevented. Contemporary surgical approaches to treat and prevent neuroma formation make use of target receptors in muscle for the regenerating axons that would otherwise form a neuroma. In targeted muscle reinnervation (TMR), the distal end of the severed nerve is connected to a motor nerve supplying a muscle, such that the regenerating axon that would otherwise form a neuroma are instead redirected into the target muscle. With the Regenerative Peripheral Nerve Interface (RPNI) technique, nerve fibers are directed to intramuscular targets by direct neurotization of small free muscle grafts (Kung et al., 2014; Woo et al., 2016; Kubiak et al., 2018). A more recently described approach involves using vascularized denervated muscle targets (VDMTs) as targets for direct neurotization (Tuffaha et al., 2020; Calotta et al., 2021). All of these approaches rest on the assumption that the axons regenerating from an injured nerve will reinnervate the denervated muscle targets rather than forming a symptomatic neuroma; however, the fate of sensory axons, and pain fibers specifically, regenerating into muscle, denervated or not, remains poorly understood. In this article, we review what is known from prior studies regarding neuroma formation and target receptors for regenerating nerves.

**TABLE 1:** Seddon-Sunderland classification of nerve injuries.

Sunderland class	Injury	Recovery prognosis	Treatment indicated
I	Neuropraxia: localized and reversible conduction blockade	Complete	No
II	Axonotmesis: axonal disruption	Complete	No
III	Axonotmesis: axonal and endoneurial sheath disruption	Incomplete, Wallerian degeneration	Medication
IV	Axonotmesis: axonal, endoneurial sheath, and perineurial sheath disruption	Wallerian degeneration, incomplete	Surgical
V	Neurotmesis: axonal, endoneurial sheath, and perineurial sheath, and epineurial sheath disruption	Wallerian degeneration, incomplete	Surgical
IV	Combination of the above injuries	Incomplete, unpredictable	Surgical

## Peripheral Nerve Regeneration (PNR)

### Nerve Anatomy

Peripheral nerves are composed of neural, vascular, and connective tissue. Nerve fibers surrounded by endoneurium form nerve fascicles that are wrapped with the perineurium sheath. Multiple fascicles arrange together to form large nerves surrounded by a dense epineurium. Nerve fibers made up of axons extend from cell bodies residing in the CNS, and they can be either unmyelinated or myelinated by Schwan cells (SCs) that wrap around the axons forming a myelin sheath (Neumeister and Winters, 2020).

### Regenerating Nerve Physiology

The cycling of neurotransmitters in neurons starts with neurotransmitter synthesis in the cell body. Kinesin and dynein motor proteins mediate anterograde and retrograde axoplasmic transport, respectively, of neurotransmitters and structural proteins. The disruption of axoplasmic flow, and the burst of action potentials initiated at the injury site are key triggers to axonal regeneration (Hanz and Fainzilber, 2006; Abe and Cavalli, 2008; Rishal and Fainzilber, 2010). Following axonal transection, a bridge of dense connective tissue and inflammatory cells forms between the proximal and the distal nerve stumps. About 24–48 h after the injury occurs, Wallerian degeneration ensues, wherein the SCs and macrophages phagocytose myelin and the distal axons degenerate (Griffin et al., 2013; Conforti et al., 2014). Furthermore, monocyte-derived macrophages migrate to the nerve bridge and add to the resident macrophages to secrete factors that induce SC dedifferentiation in the distal stump. Mostly monocyte-derived macrophages within the bridge sense the hypoxic environment surrounding a site of injury and secrete vascular endothelial growth factor A (VEGF-A), inducing angiogenesis, as well as unidentified factors that enhance SC migration to the proximal stump. The newly formed microvasculature serve as tracts that provide directionality to SC migration (Cattin et al., 2015; Cattin and Lloyd, 2016). The migrating, proliferating SCs form an empty band in the endoneurium, referred to as bands of Bungner, which guide the regrowth of axons through neurotrophic signaling (Ide, 1996; Grinsell and Keating, 2014). At the proximal end, degeneration also ensues, but stops at the first node of Ranvier (Hopkins and Slack, 1981; McQuarrie, 1985). Many axolemma sprouts form at this node and mature into a growth cone that extends through Bunger bands in response to many neurotrophic and neurite-promoting factors, ultimately reaching nervous tissue such as muscle (Lundborg et al., 1986; Zhao, 1990; Ide, 1996; Lee and Wolfe, 2000).

### Tissue Specificity in Peripheral Nerve Regeneration

Following nerve injury, numerous axonal extensions elongate at the growth cone until they connect with a receptor (Grinsell and Keating, 2014). The specific target receptors that the fibers seek to reinnervate depends on the innervated tissue type and the particular nerve fiber subtype. The resulting reinnervation pattern may or may not be identical to the original tissue and target receptors prior to injury. Specificity of sensory reinnervation of skin has been previously studied and demonstrated (Liuzzi and Tedeschi, 1991). It has been shown that transplanted dorsal root ganglia in frogs survived, entered the forelimb along with motoneurons, and regenerating sensory fibers reinnervated targets in the skin (Smith and Frank, 1987). Brushart et al. (2005) showed how electrical stimulation following the transection of the femoral trunk allowed the regenerating sensory nerve fibers from dorsal root ganglia to more specifically reinnervate the skin compared to motoneurons, providing further evidence for tissue specificity. However, sensory innervation by regenerating nerves into muscle is less specific. Koerber et al. (1995) showed that proprioceptive fibers originally innervating muscle sensory organs can reinnervate skin, although they had a propensity to reinnervate the original target tissue type. The group also found that central and peripheral stimulus adaptation properties were not perfectly restored after reinnervation, suggesting the regenerated fibers reinnervated altered reinnervation patterns (Koerber et al., 1995). Cross innervation studies in cats and rodents have demonstrated the potential to cross reinnervate tissue, where transected cutaneous nerves were shown to regenerate into the original and new cutaneous tissue, as well as skeletal muscles (Weiss and Edds, 1945; Nishimura et al., 1993). This property of cross innervation and lack of specificity of the reinnervated tissue by sensory fibers raises the possibility of alternative target receptors for regenerating sensory nerve subtypes in the setting of surgical intervention to halt neuroma formation.

### Receptor Specificity in Peripheral Nerve Regeneration

While reinnervation of receptors prevents neuroma formation, target receptor specificity depends on the nerve fiber types. It is well established in the literature that motor axons target and reinnervate motor end plates (MEPs) (Gutmann, 1945; Bader, 1980). In contrast, the target receptor for the sensory fibers in muscle tissue is still unclear. Studies have previously suggested that axonal regeneration can be deranged such that sensory cutaneous afferents innervate MEP (Allodi et al., 2012), while conflicting findings have demonstrated that sensory fibers do not reinnervate MEP (Gutmann, 1945; Weiss and Edds, 1945; Dellon et al., 1975). Possible suggested targets within muscle for sensory fibers include the Golgi tendon organ or muscle spindles (Dellon, 1991; Dellon and Aszmann, 2020). A study by Kuiken et al. (2007) found that when TMR was performed, some sensory fibers reinnervated overlying skin, although there was some evidence for deeper proprioceptive innervation in muscle as well. Earlier findings by Gutmann (1945) showed that sensory fibers did not innervate a target within the muscle, including muscle spindles. In another study, researchers connected the sensory saphenous nerve to the distal motor quadriceps nerve stump in rats, and similar to Gutmann’s study, they found that while nerve stimulation in some samples resulted in muscle contraction, this was not mediated by regenerating sensory fibers. Histologically, myelinated sensory fibers had regenerated normally into the motor stump, but the transmissible connections of regenerating fibers in muscle were only formed by a few escaped ventral root fibers (Weiss and Edds, 1945). A more recent study by Elsohemy et al. (2009) on rats demonstrated physical contact between regenerating nerves and intrafusal fibers of muscle spindles. On electrophysiologic assessment, stretching the gastrocnemius muscle elicited action potentials, indicating reinnervation of muscle spindles (Elsohemy et al., 2009). This suggests that there is a high degree of fidelity in target receptor reinnervation, but specificity is not absolute and neuroma treatment interventions can take advantage of this property. Further research is required to elucidate specific target receptors for sensory fibers regenerating into muscle tissue, and the functionality of such connections. The mechanisms of growth arrest upon contact of regenerating sensory fibers and muscle targets remains unclear.

### Free Nerve Endings Regeneration in Skin

In the skin, the neural network is widely distributed and structurally complex. Sensory nerves receive multiple modalities of input from a variety of unmyelinated free nerve endings (FNE) and specialized receptors, and conduct the stimulation through Aβ, Aδ, and C nerve fibers (Horch et al., 1977; Blais et al., 2013). FNEs are the simplest form of receptors, and the most abundant in the skin. They convey sensory information about temperature, mechanical stimuli (touch, pressure, stretch) and nociception (Horch et al., 1977; Weng et al., 2020). After injury, nerve regeneration in the skin starts from either the migration of new fibers from the wound bed, or from collateral sprouting from adjacent healthy areas (Blais et al., 2013). FNEs also exist in muscles and respond to nociceptive stimuli, and they are thought to play a role in chronic musculoskeletal pain as their density was shown to increase in an inflammatory environment (Stacey, 1969; Mense and Meyer, 1985; Reinert et al., 1998). These FNEs can grow from muscle into adjacent skin tissue. Studies have shown that skin grafts over neurotized muscle free flaps show greater degree of reinnervation with FNEs compared to non-neurotized flaps (Bayramiçli et al., 2000). Recently, specialized terminal SCs with nociceptive properties were discovered to interact with pain fibers in the skin and seem to be involved with transmission of pain signals (Abdo et al., 2019). Future studies are needed to determine whether nociceptive SCs are also present in muscle and whether they play a role in muscle reinnervation by pain fibers in the setting of TMR for neuroma treatment and prevention. Furthermore, how FNE regeneration into skin or muscle cease or what homing signals FNEs recognize in this native tissue remains to be elucidated.

### Formation of Neuromas

Following complete transection (neurotmesis), if a receptor or an endoneurial tube is not reached, regenerative axonal sprouts continue to grow blindly, producing a neuroma (Allodi et al., 2012; Grinsell and Keating, 2014). Histologically, neuromas are non-neoplastic, non-encapsulated tangled masses of axons, SCs, endoneurial cells, and perineurial cells in a dense collagenous matrix surrounded by fibroblasts (Battista and Cravioto, 1981; Cravioto and Battista, 1981; Murphey et al., 1999). Unmyelinated C fibers and thinly myelinated A-δ fibers predominate in neuromas (Cravioto and Battista, 1981; Vora et al., 2005a; Lu et al., 2018). In a histological study, Battista and Cravioto showed an increase in the unmyelinated fibers, with a ratio of unmyelinated to myelinated fibers of 20:1 (Cravioto and Battista, 1981; Lu et al., 2018). Other studies have also shown an increase in thinly myelinated fibers within neuromas (Vora et al., 2005a,b).

### Molecular Signaling in Nerve Regeneration

Although the molecular signaling in nerve regeneration had been extensively studied, signaling in disorganized regeneration leading to neuroma formation has not been clearly elucidated. The regulation of nerve regeneration is complex and multifactorial, and neurotrophic factors (NTFs) play an important role. NTFs are signaling molecules that promote peripheral nerve regeneration (PNR) and protect the injured nerve (Gordon, 2010; Shakhbazau et al., 2012). Upon injury, the injured tissue upregulates NTFs, such as nerve growth factor (NGF), glial cell line-derived neurotrophic factor (GDNF), brain-derived neurotrophic factor (BDNF), neurotrophin-3 (NT-3), neurotrophin-4 (NT-4), and insulin-like growth factor-1 (IGF-1) (Bothwell, 1997; Weng et al., 2020). Some of these NTFs exhibit specificity for certain types of nerve fibers (Allodi et al., 2012). NGF has been shown to promote regeneration of certain NGF-dependent small diameter A-δ and C fibers (Diamond et al., 1992; Weng et al., 2020). On the other hand, NT-3 promotes large diameter proprioceptive axon regeneration (Allodi et al., 2012; Liu et al., 2016). On the other hand, IGF-1 and GDNF demonstrate specificity for motor neurons and promotes axonal regeneration, motor reinnervation, and reduces muscle atrophy following peripheral nerve injury (Allodi et al., 2012; Tuffaha et al., 2016; Weng et al., 2020). The formerly described migrating, proliferating SCs form bands of Bungner and guide the regrowth of axons through neurotrophic signaling (Ide, 1996; Grinsell and Keating, 2014). Once SCs regain contact with regenerated axons, the expression of NTFs and their receptors is suppressed, creating a dynamic gradient where the highly activated SCs are located distally into the degenerated nerve stump (Mueller, 1998; Allodi et al., 2012). This gradient helps maintain the proper directionality of axonal regeneration, and after the reinnervation of the target tissue is complete, the SCs go back to a quiescent state (Taniuchi et al., 1988; Allodi et al., 2012). It is unclear if or how this gradient of NTF signaling is altered or dysregulated during the disorganized nerve regeneration in neuroma formation compared to appropriate nerve regrowth. While it remains incompletely understood, some research suggests a role for NGF, GDNF and BDNF in neuroma formation (Kryger et al., 2001; Marcol et al., 2007; Guzen et al., 2009; Valverde Guevara et al., 2014).

## Neuroma Management

### Non-surgical Treatments

Several options have been proposed for the management of neuromas, but none has been established as a superior method. Rosen and Lundborg proposed mirror therapy for relief of symptoms, proposing that amputees suffering from neuromas would benefit from manipulations of the visual system to reduce their associated pain (Rosén and Lundborg, 2005). Furthermore, evidence suggests that exercise inhibits neuroma formation and relieves allodynia through modulation of NTF expression (Tian et al., 2018). Cryotherapy, the application of low temperature to relieve pain, as well as spinal cord stimulation, which involves pulse current stimulation through electrodes implanted in the spinal cord, are other minimally invasive treatments that have also been used in the treatment of refractory neuromas (Messina et al., 2011; Rhame et al., 2011). Pharmacologic treatments with non-psychoactive cannabinoids targeting Cannabinoid receptor 2 (CB2) provides analgesic relief in painful neuromas (Anand et al., 2008; Wong et al., 2017; Wong and Cairns, 2019). Patients have also reported significant relief after steroid injections (Greenfield et al., 1984; Bennett et al., 1995). Other medications used for painful neuromas include antidepressants, anticonvulsants, alpha-receptor agonists, opioids, and lidocaine (Lu et al., 2018), although there are no clearly preferred first line agents or a standard of care.

### Surgical Treatment

Multiple surgical approaches have been developed with varying success rates, advantages, and disadvantages as summarized in Table 2. Ligation is the oldest and simplest surgical method performed, in which the proximal nerve is ligated, forming fibrous connective tissue at the ligation site (Herrmann and Gibbs, 1945). However, this method has been associated with a high failure rate and is therefore not commonly used (Guse and Moran, 2013). Burying the nerve in muscle (BIM) is a commonly used surgical technique where the proximal stump of the nerve is tucked into or under a muscle. Studies have shown that BIM successfully treated neuromas, had better functional outcomes, and prevented neuroma formation (Teneff, 1949; Dellon and Mackinnon, 1986). Other studies showed that while neuromas formed, they are sheltered from chemical and mechanical stimuli, and have decreased rates of fibrosis associated (Dellon et al., 1984). Furthermore, a study using a rat model showed that nerve implantation in vein was superior to muscles (Prasetyono et al., 2014). However, some research has shown low neuroma remission rate with this method in digital amputation (Dellon and Mackinnon, 1986).

**TABLE 2:** Advantages, disadvantages, and target receptors in surgical interventions.

Intervention	Target receptor	Advantage	Disadvantage
Simple ligation	None	Easy and quick to perform, non-sight dependent	High failure rates
BIM	None	Easy and quick to perform, non-sight dependent	Inconsistent success at preventing neuroma formation or pain resolution
Neurorrhaphy	None	Highly effective at preventing neuroma formation	Limited by availability of nerves, high technical skill required
Conduits/nerve capping	None	Can be effective, not sight dependent	Cost and availability of material
TMR	Muscle spindles, Golgi tendon organs	Highly effective at preventing neuroma formation, possible use in prosthetics control enhancement	High technical skill required, sight dependent-require recipient motor nerve stump, size mismatch, risk of neuroma in continuity, limitations on nerve size
RPNI	Muscle spindles	Highly effective at preventing neuroma formation, possible use for prosthetic control enhancement, non-sight dependent	Limitation on muscle graft size, risk of graft fibrosis and/or resorption, limitation on nerve size
VDMT	Muscle spindles	Highly effective at preventing neuroma formation, widely available recipient sights, no concerns for graft ischemia or fibrosis/resorption, use possible with large nerves	Sight dependent-vascular pedicle with muscle graft required

Another technique is neurorrhaphy—connecting two nerves to treat or prevent neuroma formation. Neurorrhaphy can be end-to-end of 2 proximal nerve stumps, or end-to-side *via* an epineural window (Gorkisch et al., 1984; Aszmann et al., 2003; Ayan et al., 2007); if only one nerve is available, the nerve can be split into two fascicles of equal size (Ives et al., 2018). Studies show 94–100% of patients report improvement or resolution of pain (Kon and Bloem, 1987; Barbera and Albert-Pamplo, 1993; Al-Qattan, 2000; Boroumand et al., 2015). Unfortunately, this technique is limited by nerve availability, and demands a high degree of technical difficulty that may produce unreliable outcomes with less experienced surgeons (Ives et al., 2018).

A newly emerging treatment method for neuromas involves capping the nerve. The goal of this approach is to inhibit growth and progression of neuroma formation by inhibiting nerve regeneration and neuroma formation (Yao et al., 2017; Lu et al., 2018). A variety of materials have been used to create nerve conduits, such as veins or epineurium, as well as synthetic materials including silicone, collagen, or chondroitin sulfate (Zuo et al., 1998; Sakai et al., 2005; Okuda et al., 2006; Galeano et al., 2009). Acellular nerve allografts have also been used in nerve capping and were found to reduce axon regeneration (Hong et al., 2019). Onode et al. (2020) found bioabsorbable conduits effective at treating neuroma-induced neuropathic pain, with prevention of perineural scar formation and neuroinflammation around the nerve stump in rat models. With both acellular nerve allografts and bioabsorbable conduits, studies showed that the length of the conduit played an important role in the efficacy of the treatment, with longer conduits better able to inhibit axonal regeneration (Hong et al., 2019; Onode et al., 2020).

Targeted muscle reinnervation (TMR) is a technique that was initially devised to enhance intuitive prosthesis control, that has since been adopted for treatment of neuromas (Pierrie et al., 2019). It involves rerouting the proximal end of cut sensory nerve stumps into the distal end of a newly divided nearby motor nerve branch (Hijjawi et al., 2006; Bowen et al., 2019; Fracol et al., 2020). The effectiveness of TMR as a treatment for neuromas and associated neuropathic pain has been well corroborated in the literature (Souza et al., 2014; Bowen et al., 2019; Dumanian et al., 2019; Salminger et al., 2019). In a randomized control trial in 28 patients standard BIM with TMR, the longitudinal mixed model analysis revealed a significant difference at 1-year post-op, with greater relief in the TMR arm compared with standard BIM (Dumanian et al., 2019). Nevertheless, possible size mismatch between the two ends poses a concern for axonal escape and formation of neuroma at the coaptation site (Mavrogenis et al., 2008; Chappell et al., 2020).

Regenerative peripheral nerve interface (RPNI) is another method which was initially designed for prosthetic control and later found to be effective in treating neuroma (Kung et al., 2014; Woo et al., 2016; Kubiak et al., 2018). This approach involves denervated free muscle grafts that provide physiological targets for peripheral nerve ingrowth (Woo et al., 2016). Some studies have shown RPNI to be effective at treating and preventing neuroma formation (Woo et al., 2016; Kubiak et al., 2019). A study by Woo et al. (2016) showed 71% of patients reported reduction in neuroma pain and 53% reduction in phantom pain. Furthermore, 75% of patients were satisfied or highly satisfied, and patients reported decreased (56%) or stable (44%) analgesic use (Woo et al., 2016). In a matched case-controlled retrospective study by Kubiak et al. (2019), RPNI effectively prevented neuroma formation (0 vs. 13.3%, *p* = 0.026) and fewer patients reported phantom pain (51.1 vs. 91.1%, *p* < 0.0001) compared to controls. Unlike TMR, RPNI does not require the denervation of residual muscles or sacrificing a motor nerve. It is also efficient, and doesn’t require tedious dissection to isolate motor nerves as in TMR (Santosa et al., 2020). However, RPNI requires devascularized muscle grafts sustained by diffusion of nutrients. Therefore it must be appropriately sized to allow for graft revascularization without tissue necrosis (Ives et al., 2018), and may limit the size of nerve used. Denervated muscle tissue with limited nutrient supply is also susceptible to fibrosis which can prevent proper reinnervation (Lee and Wolfe, 2000).

To circumvent the problem of nutrient diffusion, an RPNI can be designed with a vascular pedicle. After resection of the nerve, and deflation of a tourniquet if applicable, a nearby arterial branch to muscle is identified and confirmed with Doppler, and a small muscular flap is dissected free of its surroundings. A nerve stimulator is used to confirm denervation, and this small muscle flap is used similarly to an RPNI (Tuffaha et al., 2020). This technique provides vascularized, denervated muscle targets (VDMTs), which can be used for larger peripheral nerves, and do not suffer from ischemia-induced fibrosis and resorption (Calotta et al., 2021). The technique has been described in the upper extremity and head/neck; and can be adapted to nearly any site, allowing easy local transposition.

**FIGURE 1:**
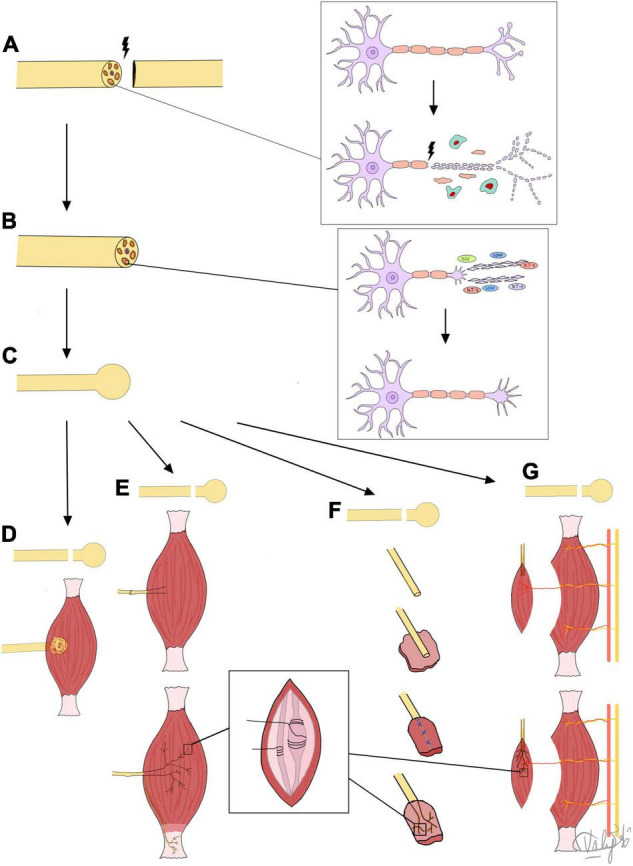
Summary of mechanisms of nerve regeneration, neuroma formation, and target receptors for regenerating fibers. **(A)** Nerve injury leads to Wallerian degeneration of nerve fiber back to first node of Ranvier. **(B)** Nerve fiber regeneration by axolemma sprouting, and growth cone and lamellipodia/filopodia formation under influence of NTFs. **(C)** Formed neuroma treated by: **(D)** BIM: excision and nerve tuck under muscle, neuroma reforms but is protected from physical and chemical stimuli or **(E)** TMR: sensory nerves regenerate into coapted motor nerve stump and into muscle to reinnervate muscle spindles and Golgi tendon organs to prevent neuroma formation, or **(F)** RPNI or **(G)** VDMT: sensory nerves directly reinnervate muscle spindles in small free muscle grafts (RPNI) or vascularized, denervated portions of muscle (VDMT) to prevent neuroma formation.

Figure 1 summarizes the mechanisms of PNR, neuroma formation and all three surgical treatment methods in TMR, RPNI, and VDMT that are based on the guided nerve regeneration to novel target receptors in muscle tissue. New approaches have sought to take advantage of a combination of the aforementioned techniques. For example, Valerio et al. (2020) reviewed a case series of 119 patients that underwent simultaneous TMR and VDMT (referred to here as “vascularized pedicled RPNI”). Only one patient developed a neuroma and required a second TMR/vRPNI (Valerio et al., 2020).

Although multiple surgeries are viable options for neuroma treatment, there is no clear superior method, and comparative data are lacking (Elliot, 2014; Ives et al., 2018; Santosa et al., 2020). This is, in part, due to limitations of current research supporting each method, including relatively short follow-up, small sample sizes, lack of control groups, and the use of non-validated outcome measures (Ives et al., 2018).

## Conclusion

Painful neuromas are problematic, and can lead to disability, loss of productivity and decreased quality of life, in addition to the financial burden they place on healthcare systems. NTFs, which promote and guide PNR, are believed to play an important role in neuroma formation, but the molecular mechanisms of neuroma formation are not fully understood. Although physical contact between regenerating nerves and their target receptors arrests nerve regeneration and neuroma formation, the mechanisms and signaling pathways causing this arrest remain to be elucidated. Moreover the target receptors within the muscle are still not completely clear, though some research provides evidence for muscle spindle reinnervation by sensory fibers. Better understanding of such mechanisms can provide an insight into treatment of neuromas.

## Future Directions

Currently, surgical management remains the mainstay of definitive treatment of neuromas. TMR, RPNI, and VDMT are surgical approaches that take advantage of introducing target receptors to regenerating sensory nerves in denervated muscle tissue. Many other surgical and non-surgical treatments for neuromas are available, but more comparative outcomes research is required to establish the best approach to neuroma treatment. Furthermore, studies elucidating the molecular signaling factors and signaling cascades downstream can provide an insight for future therapies. The study of the molecular factors that represent a homing signal to arrest nerve regeneration upon contact with target receptors can help develop therapeutic agonists to prevent neuroma formation by signal mimicking. Mapping downstream signaling cascades can provide ideas for molecular interventions on the intracellular level. Finally, understanding the molecular mechanisms of organized nerve regeneration and how they differ from signaling in disorganized nerve regeneration may help us develop therapeutics that reorganize and enhance nerve fiber regeneration while avoiding neuroma formation.

## Author Contributions

FS conducted the literature search and led manuscript planning, design, writing, and the work overall. VS illustrated Figure 1. AA contributed to the manuscript writing and editing. ST supervised manuscript planning, design, writing, editing, and the work overall. All authors contributed to the article and approved the submitted version.

## Conflict of Interest

The authors declare that the research was conducted in the absence of any commercial or financial relationships that could be construed as a potential conflict of interest.

## Publisher’s Note

All claims expressed in this article are solely those of the authors and do not necessarily represent those of their affiliated organizations, or those of the publisher, the editors and the reviewers. Any product that may be evaluated in this article, or claim that may be made by its manufacturer, is not guaranteed or endorsed by the publisher.
